# Understanding the Importance of Context: A Qualitative Study of a Location-Based Exergame to Enhance School Childrens Physical Activity

**DOI:** 10.1371/journal.pone.0160927

**Published:** 2016-08-22

**Authors:** Judy Robertson, Ruth Jepson, Andrew Macvean, Stuart Gray

**Affiliations:** 1 Moray House School of Education, University of Edinburgh, Edinburgh, Scotland, United Kingdom; 2 Scottish Collaboration for Public Health Research and Policy, University of Edinburgh, Edinburgh, Scotland, United Kingdom; 3 Computer Science, Heriot-Watt University, Edinburgh, Scotland, United Kingdom; Pennington Biomedical Research Center, UNITED STATES

## Abstract

Many public health interventions are less effective than expected in ‘real life settings’, yet little work is undertaken to understand the reasons why. The effectiveness of complex public health interventions can often be traced back to a robust programme theory (how and why an intervention brings about a change in outcome(s)) and assumptions that are made about the context in which it is implemented. Understanding whether effectiveness (or lack thereof) is due to the intervention or the context is hugely helpful in decisions about whether to a) modify the intervention; b) modify the context; c) stop providing the intervention. Exergames–also known as Active Video Games or AVGS–are video games which use the player's bodily movements as input and have potential to increase physical activity in children. However, the results of a recent pilot randomised controlled trial (RCT) of a location-based exergame (FitQuest) in a school setting were inconclusive; no significant effect was detected for any of the outcome measures. The aim of this study was to explore whether the programme theory for FitQuest was correct with respect to how and why it would change children’s perceptions of physical activity (PA) and exercise self-efficacy in the school setting. A further aim was to investigate the features of the school setting (context) that may impact on FitQuest’s implementation and effectiveness. Qualitative data (gathered during the RCT) were gathered from interviews with teachers and children, and observation of sessions using FitQuest. Thematic analysis indicated that whilst children enjoyed playing the game, engaged with goal setting within the game context and undertook low to vigorous physical activity, there were significant contextual factors that prevented it from being played as often as intended. These included environmental factors (e.g. size of the playground), school factors (cancellations due to other activities), school technology policy (rules relating to mobile phone usage) and teacher factors (engagement with the intervention). A revised logic model for the FitQuest intervention indicates how both the design of exergame technology (intervention) and features of the school environment (context) could be improved to increase chances of effectiveness in the future.

## Introduction

Physical inactivity among children is a serious public health concern. A lack of physical activity (PA) has been described as one of the most important public health problems of the 21^st^ century [1]. Physical inactivity is one of the top ten leading risk factor for global mortality; as a result, World Health Organisation Member States have agreed to reduce insufficient physical activity by 10% by 2025[2]. At present only 23% of school aged children across Europe achieve recommended levels (60 minutes of moderate- to vigorous-intensity physical activity daily) of physical activity[3]. This has serious public health implications since low PA levels are associated with poor health, including obesity, in young people. Encouraging young people to adopt healthy PA habits can help to “prevent chronic conditions including coronary heart disease, stroke, type 2 diabetes, cancer, obesity, mental health problems and musculoskeletal conditions”[4].

This paper evaluates a smart-phone based game which was designed to encourage children to increase their physical activity in school. The game design draws on UK guidance [4] which suggests that the main facilitators to being physically active are social and family influences; enjoyment; socialisation; and intrinsic and extrinsic rewards. Games are a popular enjoyable everyday activity for children. On average, 5–16 year olds in the UK spend an hour and a half playing games per day[5]. Advances in exertion interfaces and location based technologies could offer an opportunity to convert sedentary game playing time into physical activity.

Researchers have investigated the health benefits of a class of serious games known as exergames (also termed “active video games”) in which the players interact with the game through physical exercise. These games, which became more affordable for home use with the release of the Nintendo Wii in 2006, have become increasingly popular. In 2010, 39% of US youth played exergames at least once a week [6]. A meta-analysis of active video games reported that they effectively promote light to moderate intensity exercise [7], and a 2013 systematic review confirmed these findings and called for additional research “to determine how to capitalize on the potential of AVGs to increase physical activity.”[8]

Ultimately, the effectiveness, acceptability and sustainability of any complex public health intervention is dependent on robust, thorough, developmental work [9]. Stages in the developmental of an intervention include undertaking a needs assessment, developing a theory of change and theory of action (programme theory) and testing out the intervention to see whether it works as intended [9]]. This paper focuses on testing out the FitQuest programme theory through a qualitative evaluation of children and teachers’ experiences of using the game in a school setting. The data was gathered as part of a pilot cluster randomised controlled trial (RCT), the aim of which was to evaluate the effect of the exergame on self-efficacy towards physical activity, step count and minutes spent in physical activity. The results of the FitQuest pilot RCT were inconclusive; no significant effect was detected for any of the outcome measures (for trial results see [10]). A possible reason for the inconclusive findings is that the children used the game for less than half of the recommended time, and therefore were not exposed to the intervention as was intended. This finding in itself is interesting: *why* did teachers not use the game with their classes for the time period to which they agreed in advance? Were the assumptions in the programme theory met and what other contextual factors were at play?

The aim of this qualitative analysis is to explore the factors which influence the delivery, uptake and effectiveness of the game, and to refine the programme theory in the light of the findings. The objectives were to:

1. Explore whether the theories and assumptions that underpinned the intervention were working as intended.
2. Explore the extent to which the failure of the intervention was due to the intervention itself, or due to the context (including internal and external factors) in which it was implemented.
3. Provide learning for further modification of the intervention and refinement of the programme theory.

## FitQuest

FitQuest is a location-based exergame which is played on a mobile phone while running in the real world. It was originally designed and developed by the third author, and the version used in this study was implemented on the Android platform by the fourth author. It uses Google Maps™ and GPS technology to update the player’s location on-screen in the game world according to their location in the real world. FitQuest is designed primarily for outdoor play, due to the need for an accurate GPS signal. The game consists of eight mini-games based on a farmyard theme. The mini-games require running or walking, and are based on the game mechanics of collecting objects, evading capture by characters, chasing characters and racing against the clock. For example, in a mini-game called *Collect the Coins*, the user must run around the playground to collect all the yellow coins indicated on the map within a time limit, while avoiding the wolf character. As the player runs over the location of a coin in the real world, the phone vibrates to indicate they have successfully picked up an object. If the player collects the specified number of objects within the time limit, they win; if they fail to collect the objects in the time given, or the wolf catches them, they lose. Points are allocated according to an algorithm which rewards the player for improvements to their own previous performance (taking into account factors such as game outcome, distance covered, speed, difficulty level) rather than by comparison to benchmark standards or the performance of other players. Although FitQuest is a single player game, it is customary for pervasive games of this sort which require interaction in the real world to have an emergent social aspect. For example, the players might verbally compare points or run beside each other. However, FitQuest currently has no multi-player mini-games in which more than one player takes part in the same game at the same time.

FitQuest uses a modified form of goal setting as one of its main behaviour change techniques. Within the Taxonomy of Behaviour Change Techniques, *prompt specific goal setting* “involves detailed planning of what the person will do, including a definition of the behavior specifying frequency, intensity, or duration and specification of at least one context, that is, where, when, how, or with whom” [11]. Within FitQuest, the user sets personal game achievement goals which relate to the game reward system rather than real world behaviours such as walking for a specified number of steps or exercising at a particular intensity. The goals are to be achieved individually in the context of a FitQuest session in the school PE lesson. Thus, in this paper, the term “goal setting” refers to setting personal game achievement goals in FitQuest rather than the broader term. FitQuest logs user performance data and goals for each session. At the beginning of a session, the user selects from a series of goal types: achieve a player specified points target for the session; beat a previously set personal best session points target; play a player specified target number of mini-games during the session; win a target number of mini-games during the session; come first in the class leader-board at the end of the session; or complete all games on level 10 during the session. The player can optionally choose a difficulty level for the games at any time; increasing the difficulty level makes the opponents move more quickly, or reduces the time period during which items can be collected. The player has free choice over which mini-games to play and the order in which they are played. At the end of each mini-game the player is informed whether they won or lost and how many points they gained. They can access statistics about the distance they have covered and points history from the statistics menu at any time. They are informed when they meet a session goal.

Another aspect of the FitQuest design related to the Taxonomy of Behaviour Change Techniques is the leader-board, which is a form of *social comparison* which “facilitates observation of non-expert others’ performance” [11]. In FitQuest, the player can view a leader-board which shows the members of the class ranked by game points. Because points are awarded to an individual when they improve their previous performance, the leader-board effectively ranks the effort rather than the relative performance of class members. In previous pilot studies, we received feedback from some individuals who found that the leader-boards had a demotivating effect. Indeed, previous research has found that some adolescent girls experience negative social consequences within their peer group from social comparison; the authors recommend that a balance should be struck between competitive and self-referencing activities [12]. For these reasons, a player can opt out of the leader-board by asking the researcher to remove their name from the list. Because the leader-board is an optional game feature (and is relatively standard game design feature) we have not included as a key part of the programme theory. Development of the FitQuest Intervention

**Fig 1:**
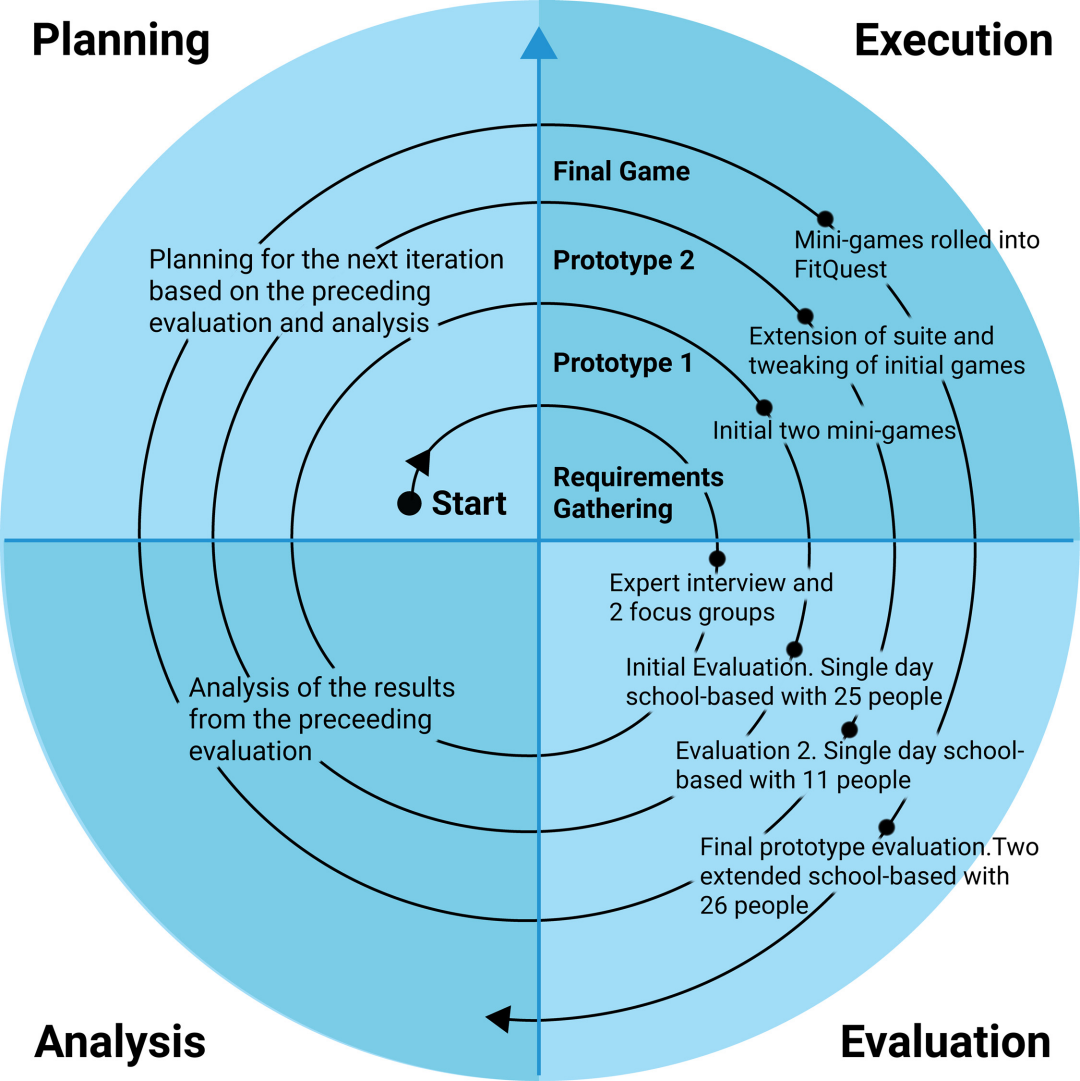
The software development lifecycle for FitQuest.

FitQuest was designed in consultation with children and teachers, using an iterative user centred design process (as depicted in Fig 1). Initial game design requirements were established from the exergame literature, and focus group interviews with children from the target demographic. Two rounds of feasibility testing were conducted with two different Physical Education (PE) classes, with feedback used to evolve and refine the game. As user feedback was positive, the final stage of the design process was to conduct two studies over a longer time period to examine the impact of the game on the user’s physical activity levels and self-efficacy. One study took place in a secondary school PE class for four sessions over 5 weeks with fourteen 14–15 year olds. The other study was in a primary school over twelve sessions (spread over 7 weeks) with twelve 11 year olds. Children reacted positively to the game, with ten of the fourteen older children and eight of the twelve younger children indicating that they would like to play the game again in future PE classes. In both studies, the children undertook light to moderate physical activity during game sessions. In depth qualitative case study analysis examined how children with different initial self-efficacy profiles interacted with the game over time and how these relate to Bandura’s social cognitive theory [13]. The findings from these two studies were used to evolve the game design requirements, namely the introduction of explicit game achievement goalsetting functionality for the children to select and configure at the start of a session.

Full details of the FitQuest user centred design process and initial evaluation results can be found in [14,15]. To build on the broadly positive, the next stage in the development and evaluation of FitQuest was to conduct a pilot RCT to gather additional evidence relating to the effectiveness of the game. Bonell et al [16] argue the case that RCTs of complex interventions in public health should focus on evaluating programme theories and mechanisms for change. A multi-case study examination of RCTs of complex public health non-technological interventions [17], highlights the importance of carefully reporting details of the personal, organisational, trial and problem contexts of the intervention to assist assessments of validity and generalisability. FitQuest is a relatively complex intervention; reliant on technology as well as teachers’ skills and commitment, and embedded in a school context. In addition, the ‘treatment’ varies considerably among participants depending on their choices during sessions. For this reason, we chose to gather qualitative data in tandem with the outcome measures of the pilot RCT in order to help us interpret the quantitative findings in the context of the programme theory.

### Programme theory

**Fig 2:**
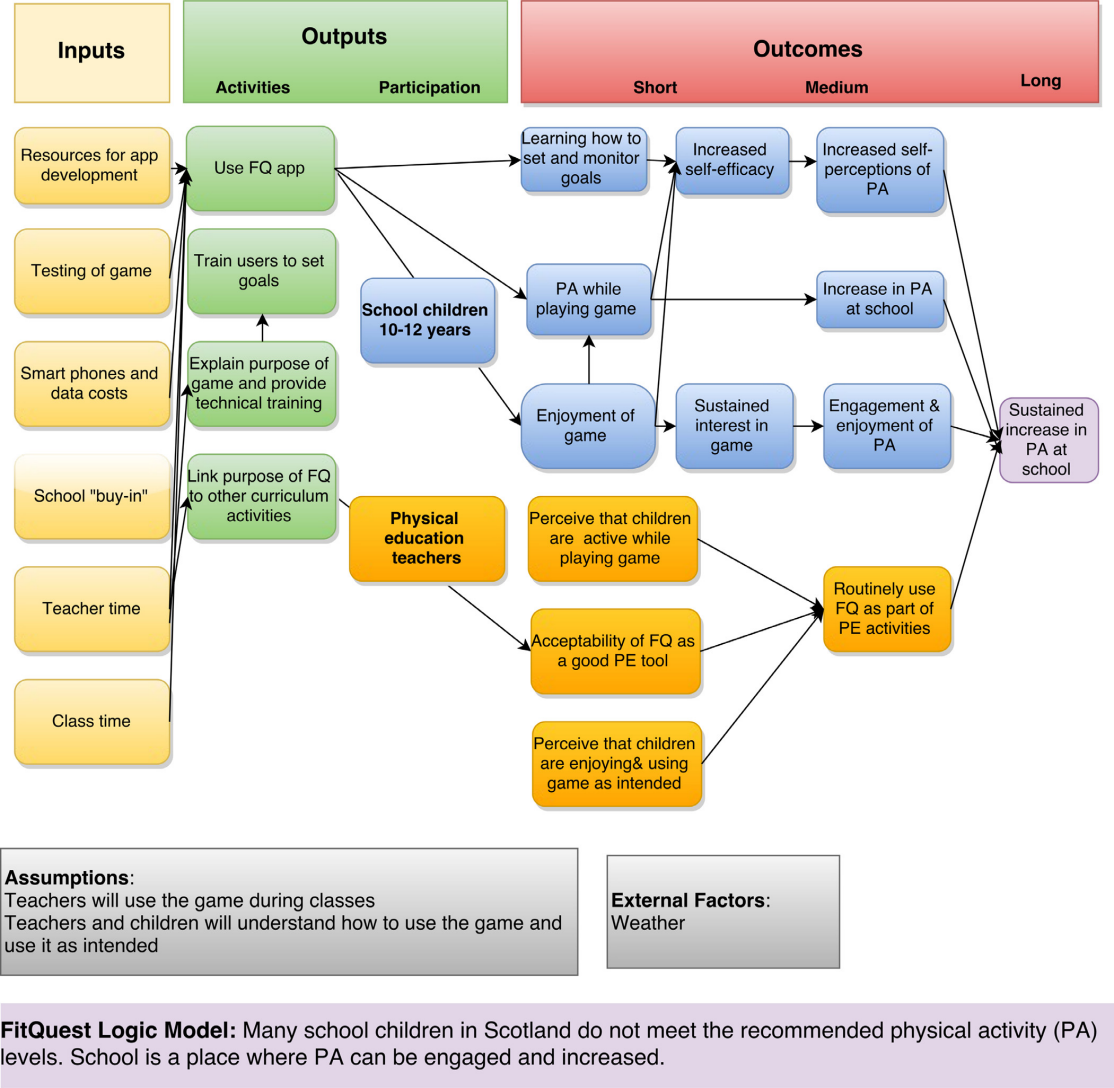
The FitQuest Logic Model.

The programme theory of FitQuest game and intervention are represented as a logic model in Fig 2. Aspects of the model relating to teachers are shown in gold, and those relating to children are shown in blue.

The main theory of change is that making physical activity a fun activity (thus increasing motivation to be physically active), in conjunction with the use of setting personal game achievement goals, will lead to sustained behaviour changes.

#### The setting

Schools were chosen as the setting in which to evaluate FitQuest as it was felt they were a conducive environment especially given that they are required to provide regular opportunities for children to undertake physical activity under the supervision of a qualified teacher (during PE) [18]. A Cochrane review of school-based physical activity programmes stated that “it is essential to promote physical activity throughout the school day during classes, lunch times, and recess, and to develop strategies to promote more efficient use of physical education class time”[19]. Therefore FitQuest intervention schools were asked to use the game during both PE classes and optionally during break-times. In all schools, FitQuest was played outdoors in the school’s playground using phones on loan from the university.

#### Key resources for the intervention (Inputs)

The inputs to the programme are the investment of resources in developing and testing the game, suites of smartphones on which to play the game, and ongoing data costs to enable communication between the phones and the university database which was used to store log files and user points. In addition, the intervention requires commitment from the schools taking part, particularly in the form of teacher time spent on attending briefing sessions and administrative tasks associated with the suite of phones. Pupils’ time spent playing the game during PE classes can also be considered as a resource.

#### Key components of the interventions (Outputs and Activities)

The outputs of the project, in terms of activities and participation were designed as follows. Ten to eleven year old children, under the instruction of PE teachers, use FitQuest during their PE lessons. While they play the game, the teacher trains the children to set personal game achievement goals. Prior to the intervention, the researcher explains the purpose of the game to the teachers, demonstrate how to use it and discuss how it might fit within the wider PE curriculum. The long-term outcome of the project was intended to be sustained increases in PA at school; in the medium term we anticipated that progress towards this long-term goal would be mediated by increases in the children’s self-efficacy and physical self-perceptions more generally, increased engagement and enjoyment of PA, and (from the teacher’s perspective) routine use of FitQuest as part of PE activities. In the short term–and as evaluated in this paper–the children’s enjoyment of the game was central to our predictions about their behaviour change. Enjoyment of the game would lead to light to moderate PA when playing the game which would lead to an increase in self-efficacy, as well as sustained interest in the game which would result in engagement and enjoyment of PA in the medium term. In addition, enthusiasm and increases in self-efficacy would result in increased PA during the school day in general, and this would remain the case after the intervention period. We also expected that children would have increased self-efficacy through learning how to set and monitor appropriate game-related goals. Teachers would perceive the children were enjoying the game and taking part appropriately and becoming more physically active. This would lead to them incorporating FitQuest into their PE lessons in the medium term.

#### Outcomes

In the logic model outcomes are categorised as short, medium and long term but could equally refer to intermediate (short to medium) and behaviour change outcomes (longer term) to acknowledge that behaviour change is usually a process which needs a number of intermediate outcomes such as self-efficacy and motivation to be changed before sustained change (in this case physical activity) occurs. Here “short term” roughly refers to the five week intervention period and post-test week, “medium term” is around two school terms or about six months (long enough for the FitQuest to be integrated into PE routines). The main short and medium term outcomes are setting personal game achievement goals and monitoring, self-efficacy, interest and enjoyment in the game.

One of the ways of motivating both children (and adults) is through enhancing self-efficacy. Self-efficacy [20] is an important psychological construct within health behaviour change; in this context it refers to a sense of confidence in one’s ability to be physically active. It has been found to play an important role in understanding and promoting health behaviour[21,22]. As the FitQuest game design allocates points for personal performance improvements [rather than in comparison to other players’ performance) and enables users to select goal types and set custom game goals that are appropriate to their ability level, it was intended to increase players’ confidence in their ability to exercise.

The pilot RCT including the qualitative study was designed to evaluate the short term outcomes in the logic model which would give indicative evidence whether the pathways to the medium and long term outcomes are plausible and the theory of change is correct. A further study would be required to evaluate these outcomes.

#### Key assumptions

The FitQuest project relied on two key assumptions: a) that teachers would give opportunities for the children to use the game during PE classes, and b) that teachers and children would understand how to use the game and play it as intended.

#### External factors that might affect success of the intervention

We anticipated the possibility that the weather would be an external factor that would influence the success of the programme. Pilot studies suggested that the children would enjoy FitQuest considerably less if the weather was poor, and that outside play sessions would be cancelled in particularly wet weather according to school policy.

## Study Design

The qualitative data reported here was gathered during a pilot cluster RCT conducted between October 2013 and April 2014 in ten state funded primary schools within a city local authority in Scotland, UK. A specialist in PE employed by the local authority identified candidate schools and sought permission from the head teachers for the study to take place in their school. Recruitment to the study began in May 2013 and finished in September 2013. There was no follow up after the intervention and post-test week. Of the 88 state funded primary schools in the local authority, ten were chosen by the specialist on the basis of being in the same geographical area of the city and having a PE specialist teacher who was willing to take part. Due to resource constraints, the study was organised in two waves: between October and December 2013, two control and two intervention schools took part (Wave 1). The remaining schools took part between January and April 2014 (Wave 2), when the study ended as planned. The schools were allocated to waves depending on their availability.

### Participants

All of the schools are in suburban areas of the same city in Scotland. Four are Roman Catholic schools [two control and two intervention), and the others are non-denominational. The schools can be rated according to the Scottish Index of Multiple Deprivation [SIMD) from the second lowest quintile [2] to the highest [5] (see Table 1). The SIMD is the official tool used by the Scottish Government to identify areas of Scotland which suffer from deprivation in terms of a combined index of employment, income, health, education, geographic access to services, crime and housing [23]. Postcodes indexed in Quintile 1 are the most deprived and those in Quintile 5 are least deprived.

**Table 1:** Information about schools.

School ID	SIMD quintile	Arm	Wave	Boys	Girls	Total
1	2	FitQuest	2	9	9	18
2	2	FitQuest	1	13	14	27
4	5	FitQuest	1	14	10	24
7	4	FitQuest	2	9	12	21
10	5	FitQuest	2	10	11	21
3	2	Control	2	4	9	13
5	5	Control	1	5	11	16
6	5	Control	2	13	17	30
8	3	Control	1	9	9	18
9	2	Control	2	14	13	27

Participants were the pupils from a complete primary seven class in the ten selected schools. If there were two classes in the same year group in intervention schools, both classes had the option to use FitQuest in the interests of fairness but only data from one class (selected in advance by the school) was included in the analysis. Similarly, control schools with two classes in the same year group collected pre- and post-test data from both classes, discarding data from one class which was decided in advance by the school.

### Delivery of the intervention in the school settings

The FitQuest project loaned each school allocated to the intervention group one suite of fifteen Samsung Galaxy Ace II phones for the duration of the project. Due to resource constraints, the children shared these phones with a partner, but each child had a personal user name and password to ensure that only their own performance data was recorded. The project paid for all data costs on the mobile phones. Each school had access to outdoor play space for FitQuest use in the form of a tarmac playground, a grassy field or an all-weather surface.

Schools in the intervention arm used FitQuest for 5 weeks during PE lessons. Pupils in Scotland have a mandatory total of two hours of PE classes per week (usually split into two 1-hour lessons, as was the case in all the schools participating). Schools undertook to use FitQuest during both of these classes for the duration of the study. However, due to the limitation in the number of mobile devices, only half of class could use FitQuest at any given time. The one-hour lessons were split into two 30-minute play sessions. Each participant, therefore, played the game for approximately one hour per week within his or her PE class, over two 30-minute sessions. Mobile phones were handed out to the children at the beginning of the session and returned to the teacher at the end of the session. The children were given a demonstration of how to use the game on the first session, and researchers provided help to individual children subsequently when necessary. During the PE lesson, when the children were not using the FitQuest system, they were given a traditional PE activity. This differed from session to session and was at the discretion of the PE teacher. Activities included football, field hockey and traditional playground games.

In addition to the two PE sessions per week, at the discretion of the classroom teachers, FitQuest could be used during school break-times. All of the required equipment was left with the classroom teacher for the duration of the evaluation, such that should they desire, children could optionally play the game during their morning or afternoon breaks. The way in which this process was managed was fully under the guidance of the classroom teacher. In school 4, the teacher created a rota so that children did not all try to take a device during the same break-time. During these sessions, FitQuest operated in much the same way as during the PE based sessions, with children free to set game goals, and construct their own session from the mini-games. Due to the organic nature of these sessions, researchers did not observe break-time use of FitQuest.

Participants in the control arm took part in the normal PE classes provided by the school for 5 weeks. These activities varied according to the school and teacher but included gymnastics, football, hockey, badminton, rounders, dancing, playground games, and basketball.

### Ethics

The study was approved by the School of Life Sciences Ethics Committee at Heriot-Watt University, Scotland. Written permission to conduct a study in the schools was given by Edinburgh City Council. Written informed consent was given by one parent/guardian and all child participants.

### Qualitative data gathering and analysis

The data gathering strategy in the study as a whole was to triangulate data between quantitative outcome measures (step count, minutes spent in moderate to vigorous physical activity (MVPA) and self-efficacy); comprehensive log file data on individual’s usage patterns; qualitative observations of the game in use and interviews with children and teachers. This analysis in this paper focusses on the observation and interview data. The purpose of the observations was to gain an overall impression of how the game was used in the physical space, and to understand emergent patterns of social interaction. It was also useful to informally track participants’ changing attitudes between sessions and to get their immediate opinions in case they were unable to remember specific incidents in the post-test interview. All observations focused on the FitQuest sessions, and not the traditional PE lessons which occurred simultaneously. The interviews at the end of the study were an opportunity for the participants to reflect on their overall experiences, particularly on goal setting and to explore behaviour which occurred during observations.

The data collection team consisted of the first, third and fourth authors who are all computer scientists who specialise in interaction design for children. The children were aware that the team were involved in the design and implementation of the software, and read information sheets which explained that the game is intended to increase physical activity. From the outset, the children were encouraged to critique the game and provide suggestions for improving it during sessions.

At least one researcher from the data collection team was present for all PE based usage of FitQuest in order to gather observational data and record very short interviews with the children as they rested between mini-games. The first author observed at Schools 1,7,10; the third author observed at all intervention schools; and the fourth author observed at Schools 2, 4, and 7. Where possible, two observers were present at each session, and observers were matched to schools according to convenience of timetabling and geographical location. As is common in research in educational settings, the role of the researcher was participant- observer in the sense that researchers provided technical assistance to the children, and interacted with them about their experiences. The researchers approached children to ask questions to aid interpretation of their behaviour, and the children frequently spontaneously approached researchers to report problems, volunteer ideas and share successes or failures. The research team did not attempt to systematically observe each individual during each session because comprehensive information for the choices and performance of each participant was continuously documented in the log files. Observations offered a unique opportunity to subjectively study the use of the system in a complex social situation. The team either dictated (unstructured) audio notes during the session, or wrote them by hand. Each researcher transcribed their own notes and added a personal reflection at the end of each session.

During the week immediately following the evaluation period, two researchers (the first and third authors) conducted 20 minute semi-structured interviews with a total of 6 pairs of children (from two of the intervention schools–schools 2 and7). The interview questions were prepared in advance and the questions can be found in the supplementary materials. One school from each wave was selected according to the availability of the classes for interview in the post-test week (convenience sampling). The children were selected using critical case sampling (a form of purposive sampling) by the researcher team after a discussion in which the two researchers who observed the sessions at the school identified which children displayed behaviour related to the aims of the study such as changes in self-efficacy or clear patterns of goal setting. Importantly, while all children exhibited behaviours of interest, we aimed for a balance between what could be described as a ‘positive’ and ‘negative’ experience, for example, by recruiting not only those that remained engaged, but also those that showed clear signs of disengagement We wanted to use the interview questions to gain more insight into these behaviours than was possible from “snapshots” of interactions during the observation sessions. We chose an equal mix of boys and girls, from children whose comments or behaviour during observation suggested that they would have negative as well as positive views. The children were interviewed in pairs in order to help them feel more comfortable. The pairing of children was aided by the class teacher, who helped ensure children were paired with a peer with whom they worked well. Interviews took place in an open-plan area outside the classroom in School 2 and in the corner of the classroom in School 7. The audio recordings of the interviews were transcribed by the researcher who recorded them.

Three teachers from two of the intervention schools, one from each wave (the class teacher and PE teacher from school 2 and the PE teacher from school 10) were also interviewed in the post-test week after school or at break time on the school premises. The interviews were audio recorded and transcribed. All teachers from the intervention schools were invited to be interviewed, but only these three were available.

A COREQ checklist with further details of the qualitative design is included in the supplementary materials (S1 Checklist).

A paper version of the Pender questionnaire [24] on exercise self-efficacy was administered to all participants in the study pre and post-test. These were completed during class time. In addition, FitQuest generated log files for every play session: time spent playing the game, number of sessions, game points, individual mini-game outcomes, difficulty level changes, user specified custom points targets and the number of each type of goal attempted and achieved were all recorded.

Thematic analysis [25]was conducted on observation notes from eighteen FitQuest sessions and semi-structured interviews from three teachers and twelve children. The analysis was both deductive (to explore issues around the underlying programme theory and associated assumptions outlined in the logic model in Fig 2) and inductive (allowing for new issues to emerge). The notes and transcripts were coded by the first author using the DeDoose qualitative analysis software [26] and a selection of the transcripts were independently coded by the second author. The themes specified before analysis were those in the logic model: game goal setting and monitoring; self-efficacy; physical activity; enjoyment; sustained interest and teachers’ opinions. The emergent themes relate to suggestions for improving the game and contextual factors. Where possible, interview and observation data has been triangulated with the self-efficacy scores and log file information from the wider RCT to facilitate deeper understanding of the participant’s situation. Once the thematic analysis was complete, the analyst considered the evidence theme by theme. She attempted to find additional evidence relating to that theme for each individual in interviews, observations, self-efficacy scores and log-files with an emphasis on tracing changes in attitudes over time, and objective evidence from log files which would enable a deeper understanding of the children’s verbal comments or behaviour. Children, teacher and school identities have been anonymised and replaced by pseudonyms throughout. The excerpts from interview and observation transcripts for each theme is included in the supplementary materials (S1 Transcripts).

## Results

### Assumptions

The assumptions in the logic model were that teachers would use the game in class, and that teachers and children would understand how to play the game and use the game as intended.

Table 2 shows the log file data on the average number of minutes spent playing the game at each school. While each child did use the game in class, they did not use it for the period of time recommended by the researchers (60 minutes per week for 5 weeks). School 1 used it for only 2 sessions (13% of the recommended time) while school 7- the school with highest usage—used it for 8 sessions (51% of the recommended time).

**Table 2:** Treatment fidelity (time spent using FitQuest in treatment schools).

		Mean time spent using FitQuest in minutes per child	Standard Deviation	Number of sessions	Proportion of recommended usage time
School ID	1	38	9	2	13%
	2	121	51	8	40%
	4	125	15	6	42%
	7	151	20	8	51%
	10	73	20	5	24%

The teachers understood how to play the game when they used it at the initial training session, although some of them did find it difficult to navigate initially. There is substantial evidence that the children understood how to play the game, and that the majority of them played it as intended. Observations confirmed that children learned how to play the game although some had initial difficulty in understanding the navigation representation. This was usually resolved by some instruction from the researchers, who explained how to use an alternative map view (with a dynamic rather than static camera) which some users prefer.

Log file data confirms observational evidence that the children understood how to play the game after the initial learning period. The lowest points score recorded in the log files across all FitQuest users was 49 which indicates that each child was able to use the game well enough to gain points. On average, the users across all schools scored 34 points per session. Short to medium term outcomes.

#### Game goal setting and monitoring

The children were able to use the goal setting features of FitQuest. They set on average 79 goals each across the duration of the project. The average success rate for these goals was around 49%. Only 3 children failed to achieve any goals, two from School 2 and one from School 4.

Observational and interview evidence indicates that the children were pleased when they achieved the goals that they set. The children would frequently exclaim aloud to their friends or the researchers when they achieved a goal, and reported feeling happy and excited when they did so

Although the game goal setting aspect was designed (at the suggestion of a PE specialist teacher) with the expectation that the teacher would help the children develop suitable goals, we did not observe this in any of the sessions. The teachers were aware of the game goal setting feature but were not usually available to advise on it during sessions as they were teaching the other half of the class. It would, however, have been possible to do so at the beginning or end of a session.

Setting and monitoring appropriate goals involving numerical calculations is potentially cognitively challenging for some children of this age, particularly in deciding what action to take after succeeding or failing in a previous goal. This is clearest in the case of the goal of setting a numerical points target. Here the child has to read the feedback from FitQuest on whether they achieved their target, recall how much time it took to achieve these points, and then reason about an appropriate response given the time remaining in the session. When asked, some children were successfully able to reason about how many points they were likely to get in a given time, although others were less realistic, for example in trying to achieve more points than they had earned in the first twenty minutes of a session in the final five minutes.

In the interviews, the children mostly demonstrated that they could successfully reason about how to appropriately change their goals under success or failure of the previous goal. When asked what they would do if they achieved goal, they replied they would set the goal to be slightly higher the next time, and that if they failed to achieve a goal they would reduce their target for the next time until they were able to achieve it.

Some children appeared to particularly appreciate the goal setting feature. When asked what she liked about the game, Lesley replied that she liked the sense of achievement related to goals and points.

Analysis of the quantitative self-efficacy and log file data enables us to explore Lesley’s patterns of use in more depth. Lesley’s pre-test self-efficacy score was below the median but increased by 7 by the post-test. For context, the maximum self-efficacy increase across control and experimental groups was 9. The Pender questionnaire is a likert scale, so moving one’s answer upwards one position in each of the 8 questions (e.g. from neutral to agree) would result in a numerical increase of 8, according to the scoring scheme. The maximum possible score on the questionnaire is 32. She achieved 55% of the custom points goals she set (in comparison to an average of 49% across all FitQuest users). Her steady accumulation of points put her in the 97^th^ percentile of total points scores across the FitQuest sample. In Lesley’s case, the pathway from game goal setting to increased self-efficacy appears to have worked as intended.

James also experienced success in goal setting and an increase in self-efficacy. He demonstrated a cautious attitude to goal setting, setting targets far lower than his previous best performances in order to be sure he achieved his goal.

James started with a self-efficacy score below the median in the pre-test but raised it by 8. He achieved 75% of the goals he attempted. James’ seemingly cautious attitude to goal setting paid off with regular experiences of success, which may have resulted in his self-efficacy increase.

When asked why he set the ambitious goal of completing the games on the hardest difficulty level, one of the top performing children spoke of the fun he gained by repeatedly achieving a challenging target. This demonstrates the link between high self-efficacy, enjoyment and PA effort.

Much of the goal setting in early sessions was centred around the leader-board, although observations suggest that the emphasis moved more towards personal best goals for some children, and other children reported being indifferent to the leader-board. In School 7, the leader-board appeared to have a negative impact. The children in all schools were told that they could opt out of the leader-board (i.e. prevent their name from appearing on the ranked score list which was available to their classmates) if they wished. This resulted in several children at School 4 swapping in and out of the leader-board (no children at other schools did so). For example, in session 2, Simon asked to be removed from the leader-board and in session 3 the researcher observed that Simon was very concerned to keep his points private. In the last session he asked other where they were on the leader-board before coming to the conclusion that he was the best in the class. He then asked to be reinstated because he thought he had most points. Unfortunately, Simon was mistaken and he did not have the most points: in fact he was in the lowest quartile for points in his school. Simon achieved 38% of his target goals. He never selected the “top the leader-board” goal, focussing instead on personal best and custom points score goals. Although Simon started with self-efficacy above the median, his self-efficacy score at post-test had decreased by 8 (the maximum decrease in the sample group was -9). It is possible that his self-efficacy was negatively impacted by his ambivalence to the leader-board, in conjunction with the fact that his points score was lower than his peers. There was a strong competitive atmosphere within the class at this school; some children teased their peers for low rankings in the leader-board and the researcher’s notes indicate that Simon was the recipient of such teasing in an early session.

Interestingly, some individuals maintained their self-efficacy in spite of repeated failures to meet self-set targets. For example, Saanjh achieved only 3 goals of the 49 he set himself (largely because he mostly chose the ambitious goal to become first on the leader-board). His self-efficacy score increased by 1 during the project and his interview indicates pleasure in achieving “quite a few” of his goals; it would appear that he did not find the low rate of overall goal success particularly demoralising. Perhaps for some individuals the enjoyment of a small number of successes is motivating. The leader-board also resulted in direct rivalry between particular children, as indicated by participants who explained to the researcher that their leader-board goal was not to come first but to achieve a higher rank than a specific individual. One child reported working up the leader-board rankings in order, trying systematically to beat the child in the place above him each time.

A position at the top of the leader-board did not always have a positive effect on motivation. Researchers’ notes indicate that Evan, who had been at the top of the leader-board in his school in the first two sessions, appeared to reduce his effort during session 3, while closely monitoring rivals lower down the leader-board. Evan had initially high self-efficacy which had increased by 1 by the post-test. Evan did win the leader-board for his school with 583 points. The maximum points across all FitQuest schools was 996 however, so he had reached only a local maximum of performance. It would be unfortunate if the leader-board has the effect of placing a ceiling on effort in some individuals.

In summary, the observations and interviews (triangulated with self-efficacy and log-file data) suggest that goal setting did have a positive impact on motivation, and despite the potential cognitive challenge, some children were capable of appropriately adjusting their goals in response to success or failure. However, the goal of topping the leader-board was not a consistently effective motivator across the participants.

#### Self-efficacy

In the logic model, mastery of goals, enjoyment of the game, and taking part in PA while playing should all lead to an increase in self-efficacy. These relationships are not necessarily linear, but build upon one another (e.g. mastery of goals can lead to greater enjoyment which can then increase a feeling of self-efficacy). Some evidence relating to the relationship between self-efficacy and the game emerged from the interviews and observations. At interview, some children reported being *“happy”* and *“proud”* of what they achieved, including some who did not usually consider themselves as “fit” or “fast at running”.For these individuals, putting in effort at FitQuest became enjoyable and motivating and provided them with evidence that they could successfully participate in PA. There is also some evidence that the children could persist in exercising with FitQuest even if they were feeling tired or were not in the mood (both statements in the Pender self-efficacy questionnaire used in the RCT as an outcome measure).

For some children, self-efficacy could be reduced by failure to meet goals. Isabel commented that she would feel sad and consider herself to be “not sporty” if she didn’t meet her goal. Another child associated a low rank in the leader-board with a reduction in confidence.

A lack of self-efficacy was occasionally associated with a lack of motivation to keep playing FitQuest. Some children only chose to play mini-games they were good at, or would become demotivated if they were unable to succeed in their goal. A class teacher at School 4 commented that one child who appeared to only play games where he would do very well, became demotivated when he realised that he was not first on the leader-board and “stopped putting in effort as an excuse”.

This pattern of behaviour is indicative of low self-efficacy. This child became disruptive to other class members due to his lack of engagement with FitQuest.

#### Physical activity

The ultimate aim of the intervention was to increase physical activity, via one of the pathways described in the previous sections. Evidence from observation notes indicates that many of the children were moderately to vigorously active while playing FitQuest.

The children’s play was often characterised by cycles of fast running as they played a mini-game, followed by a short rest (often standing next to friends, comparing points, discussing game tactics and planning). A small number of the children appeared to be expending a great deal of effort: as exhibited by breathing very heavily, or lying on the ground at the end of the session. Some children exercised steadily at lower intensity throughout the session. Others spent longer periods resting, particularly if their friends were also doing so as they enjoyed chatting before choosing the next game to play.

Some of the children identified that their fitness level was improving. Brendan associated the *Escape the Wolf* mini-game with improving his stamina and Douglas felt that playing FitQuest improved his speed, stamina and performance in other sports. Linda, who initially told the researchers that she could not run fast, reported at session 4 that she was “pushing herself to do lots of running” and that her speed had increased.

Not all of the children were active in every session. Sometimes the children appeared to be distracted from running by social interactions. In the initial sessions, difficulty in understanding the map representation in the game interface would cause the children to walk slowly or stop. The children did appear to tire at the end of sessions and reduced their PA intensity accordingly. On one occasion the whole class appeared to be tired because they had been skipping in the lesson immediately prior to the session.

#### Enjoyment of game

Evidence from observations and interviews with children and teachers indicate that the children generally did enjoy playing the game. At interview, the children spoke positively of the game, describing it as “fun” and “awesome”. They were typically very excited to take part and the researchers were often asked if it was possible to buy the game for use at home. The children were often very engaged as demonstrated by reluctance to stop when the session ended. At School 1, the children were motivated to play through heavy rain to gain more points in their last session and were disappointed when the adults insisted they return indoors.

Often the interviewees linked their enjoyment to their physical activity without being explicitly asked, for example by saying it was “good exercise” or made them “out of breath”.

A small number of individuals, however, did not enjoy FitQuest:. During session 2, Dominic (School 2) complained that he found FitQuest boring Dominic’s total points score was in the lowest quartile in his school and he achieved none of the goals he set, although his self-efficacy started and remained high. This example confirms the pathway in the logic model through which effort in physical activity (as estimated by points in this example) is moderated by enjoyment in the sense that his lack of enjoyment of FitQuest was apparently related to lack of effort when playing it.

A small number of children became frustrated if they did not understand how to play the game and this impacted their enjoyment, as illustrated in the following excerpt. For example, researcher’s notes at School 10 record that Lachlan was annoyed because he was confused about how to navigate in the game. After a demonstration by the researcher, Lachlan did master how to play the game and went on the achieve 14 out of 19 of the goals he set.

#### Sustained interest in game

There was mixed evidence from the qualitative data about how the children’s interest towards the game changed. In School 2, the only one in which teachers offered children the option to use the game at break times, the children appeared to tire of the game after several sessions. The PE teacher at School 2 commented that although initially “huge”, the children’s motivation waned after two weeks. This is consistent with the views of Maurice and Aakil (School 2) who said that the game got boring after they had played them many times, and is also reported in observation notes.

The PE teacher at School 10 noted a similar pattern in that the children’s interested “tailed off” once they got used to it. She also commented that when the activity for the other half of the class was football in the last week, the children generally were keener to play football, but not all of them. She noted that Craig, who in her view had previously been “lazy”, kept up sustained interest in FitQuest. The teacher was correct in her estimation of Craig’s effort. He achieved 70% of his goals, was in the highest quartile for points in his class, and increased his self-efficacy score by 4 during the project.

In School 7 (the school where on average children played the game for longest), a different general pattern emerged. Excerpts from the mini-interviews during observation sessions appear to show that the children liked the game more as the sessions progressed. For some of these children, their increase in enjoyment as the weeks progressed was related to mastery. Linda, Emma and Robert reported that they enjoyed it more once they understood how to play the game and found it easier to play. At School 4, observation notes suggest that there was not a general decrease in motivation as the project progressed, although a small number of individual children became bored. In the remaining school, the children were extremely keen to keep playing after the last session–this may well be because this was in fact only their third opportunity to play the game at all.

In summary, although some children became bored of the game after several sessions, others enjoyed the game more as time went on demonstrating sustained interest.

#### Teachers’ opinions about FitQuest as a tool for PE

Both the PE teachers that we interviewed were positive about the intensity of the children’s physical activity. The PE teacher from School 2 noticed that the children were running as fast as they could for 30 seconds (“strenuous PA”) followed by a minute of recovery. The PE teacher from School 10 considered that FitQuest was “very good for their fitness*”*. and that the children exercised more than they would if they had been asked to do a traditional running activity.

The teachers also commented positively on the children’s enjoyment: The PE teacher from School 10 reflected on some of the reasons why the children enjoyed the game including immediate feedback to acknowledge their efforts from points and the leader-board.

However, the teachers’ opinions on the acceptability of FitQuest as a good PE tool were more cautious. The School 2 PE teacher was initially “sceptical” about the project because he questioned the role of technology in a PE class. However, he valued the children’s increased PA while using FitQuest and was “very happy” to have taken part in the project. This teacher also praised the benefits of *“authentic learning”* within the project, commenting that playing games on phones is an activity which children would naturally do as part of their everyday lives.

The PE teacher at School 10 spoke positively of the children’s technological skills which enabled them to learn how to play the game quickly. She said that the children “took ownership” of the game and could operate it by themselves. She recommended that FitQuest should have a stronger component of social play to fit with the current PE curriculum, particularly team work. This teacher also expressed reservations about her own technology skills and how she didn’t feel able to help the children. She felt there might be logistical barriers associated with the hardware, in terms of maintaining it and making sure the devices were charged and ready to use for each session. The class teacher in School 2 had already worked out a solution for some of the anticipated problems, by delegating responsibility of the devices to the children.

The School 10 teacher identified that lack of budget for schools to buy phones could be a problem in the future. She suggested that it would be beneficial for the children to share phones to reduce the cost, or for a group of schools to pool resources. In terms of how FitQuest could be incorporated into time planning across the year, she suggested that it could be used for shorter blocks of time, interspersed with other activities.

### Inductive (emergent) findings

#### Suggestions for improving the game

The teachers had suggestions for improving FitQuest to make it more suitable for use in PE. The School 10 teacher suggested a “multi-layering” approach where she would introduce additional physical skills to be used while playing the game, such as dribbling a football, or simultaneously trying to kick balls into a net. The PE teacher in School 2 suggested that the teacher could do more direct instruction about the game to ensure that all the children knew how to use each feature.

In order to sustain the pace of the game throughout the session, he suggested that the pace should be determined by the game rather than player choice, and that the game should set the challenges.He also suggested including hidden games to sustain engagement and introducing a wider variety of game themes including cops and robbers. Some children also suggested increasing the variety of games available. The children suggested some technical improvements for FitQuest including improving the user interface and reducing the GPS latency.

FitQuest was designed to be social in the open ended fashion of a pervasive game. It was therefore an open question how social interaction itself would evolve and how it would influence player experience. It was apparent throughout the observations and interviews that social play was a great influence on the success of the project, and was explicitly encouraged by some teachers. Although FitQuest is a single player game, the children often used it socially by comparing scores with friends, running alongside friends as they played the same game, and clustering together during rests. A class teacher (in the school where the game was available to children during breaks) said that FitQuest gave solitary children a purpose during break time:. She considered it positive that the game gave such children guidance, challenge and non-player characters (such as the wolf) to play against.

The idea of a multi-player game where players could interact with real world friends in the game space was often suggested when we asked the children how we could improve the game. They also suggested including team games and team competitions using the leader-board. Brendan suggested modifying the leader-board goal type so that the children could choose to be in the “top 10” or similar.

However, social interaction did not always have a positive impact on motivation. There were influential individuals at three of the schools whose lack of motivation and negative comments discouraged and distracted their peers from engaging. When these pupils were absent, their peers appeared to enjoy the game and expend more effort. The leader-board also had mixed effects on motivation due to the element of competition On balance, however, social interaction acted as a facilitator more often than as a barrier.

#### Contextual factors

Some additional barriers and facilitators emerged during the analysis of the qualitative data. In the early sessions, technical glitches caused by poor GPS signals were frustrating to some of the children, and some had initial difficulties learning how to navigate. For most children, these problems resolved within the first two sessions, but it was a particular problem for children who feared they would lose face by appearing to their classmates as incompetent with technology or as *“*slow runners*”*.

Lack of space in the playground was also a barrier. The smallest playground was at School 10, where the GPS signal was shadowed by the school building. This was very frustrating to the children because it meant that the position of their characters updated slowly, and that in-game objects sometimes were inaccessible because their location was randomly generated at the start of each game.

It was envisaged that the schools would give the children the opportunity to play the game in the playground at break times. In practice, only School 2 facilitated this. One teacher mentioned a policy of no mobile phones in the school playground and did not want to confuse the children by making an exception for the project.

An emergent facilitator is the presence of an enthusiastic adult with whom the children can share their progress. In School 2, the PE and class teachers took great interest in progress, whereas in at School 1 and School 7, the children were keen to discuss their experiences with the researchers.

## Discussion

A review of ecological models in health promotion notes that research in PA has moved towards more ecological approaches in the last twenty years[27]. Such approaches consider that behaviour change interventions should address the settings and social contexts which shape behaviour as well as the cognitive and affective processes within an individual. However, exergame research has largely taken place in lab-based studies or clinical interventions without consideration of social and contextual factors [8]. A notable exception is the American Horse Power Challenge (AHPC) which used an ecological approach to consider the wider social-technical environment of a physical activity game deployed in sixty-one schools across the United States[28]. The findings of the present study complement and extend the AHPC results; although they are generally consistent, the differences in the game mechanics and technology platforms used in the studies give insight into the impact of different design features. For example, the AHPC game did not allow users to set their own game goals, and it promoted competition between schools rather than individuals. Exergames should not be considered as black-boxes which can be used interchangeably in interventions- the underlying theories of behavioural change and the ways in which they are implemented in the game design will impact patterns of user behaviour.

### Reflections on behaviour change techniques used in the game design

#### Goal setting and monitoring

The qualitative findings indicate that game goal setting and monitoring was motivating for the children, and interview and observation data show that the children in general were capable of adjusting their targets appropriately according to success or failure. This suggests that this aspect of the underlying programme theory is correct. However, the way it was implemented in the game could be improved.

In FitQuest, the game goals are self-defined rather than specified by the researchers. At the suggestion of an expert PE teacher, users choose between goal types and set numerical targets such as number of points to earn in a session, or the number of games they aim to win. The rationale for this is that within the context of a PE lesson where FitQuest is used, learners should actively “… monitor their bodily responses, consider alternatives for increasing or decreasing MVPA, and set and reach reasonable goals as they construct meaning from their experience”[29]. By developing the cognitive and affective skills related to setting and monitoring goals, learners will be better able to be physically active on their own in the future, without the presence of a teacher, or indeed technology. The FitQuest design could be improved by actively assisting young learners to develop the necessary meta-cognitive skills for goal setting and monitoring. For example, it could initially set an appropriate default goal and give feedback on how realistic user-defined goals are in the light of previous performance.

Unlike previous goal-setting studies for health behaviour change in children[30,31], the goals in the FitQuest study were related to the game points rather than real world behaviours such as vegetable consumption or step-count. The goal setting feature in FitQuest could potentially be improved by assisting the children in mapping their performance within the game to real world indicators (e.g. displaying game points with the equivalent step counts or minutes exercised on the same screen). This would then enable the children to reason about how to continue to achieve their goals at home after the FitQuest session is complete or work towards achieving them in the next session.

#### Social comparison

The leader-board did not have an entirely positive impact on the children’s motivation; this was related to the interplay of an individual’s self-efficacy with their social relationships with their peers. This is consistent with a literature review on gamification which suggested that different players experience different motivational affordances (including leader-boards) differently, and that the context of use is an important factor[32]. The findings of the AHPC study illustrate the impact of a variant of social comparison: the competition took place at school level rather than individual level. Unfortunately, this led to over-competitive and unconstructive rivalry [28] between some schools, depending on geographical context and existing rivalries. Similarly, other studies of social comparison in apps to promote physical activity are ambivalent, depending on the individual differences in attitudes to leader-boards: a programme of research to systematically investigate the motivational affordances of such techniques in different contexts is required[33].

The results of the present study indicate that although some individuals initially focussed on the leader-board goal rather than self-referencing goals, this did fluctuate over time. In later sessions, some users became disengaged with the leader-board and found it demotivating, while others shifted their focus to other goal-types. This reinforces the point that if leader-boards are to be used for behaviour change, which takes place over an extended period of time, longer term motivational effects should be considered–for example regularly appearing last in the leader-board might eventually be damaging to self-efficacy. The range of attitudes to the leader-boards, and the fact that many children chose to focus on goals which are not related to the leader-board also support Zuckerman and Gal-Oz’s recommendation that gamification elements such as social comparison should be tailored according to the personal characteristics and preferences of each user rather than appearing as a standard feature for all players [33].

### Logic model revisited

The findings from this study confirm that the context in which an intervention is implemented is likely to be as important to the effectiveness as the intervention itself. Indeed contextual barriers can significantly inhibit the effectiveness of a promising intervention. Interventions can be viewed as events in a system and they are best understood by employing systems theory. “A system is a set of actors, activities and settings that are directly or indirectly perceived to have influence in or be affected by a given problem situation”[34]. Within a system, an intervention exerts its influence by changing relationships, displacing existing activities and redistributing and transforming resources[35]. The system in this study was the school system which had many actors (e.g. teachers and head teachers), external events and structures (e.g. size of the playground) which influenced the delivery of the intervention and hence the programme theory was found to lacking in some key aspects.

**Fig 3:**
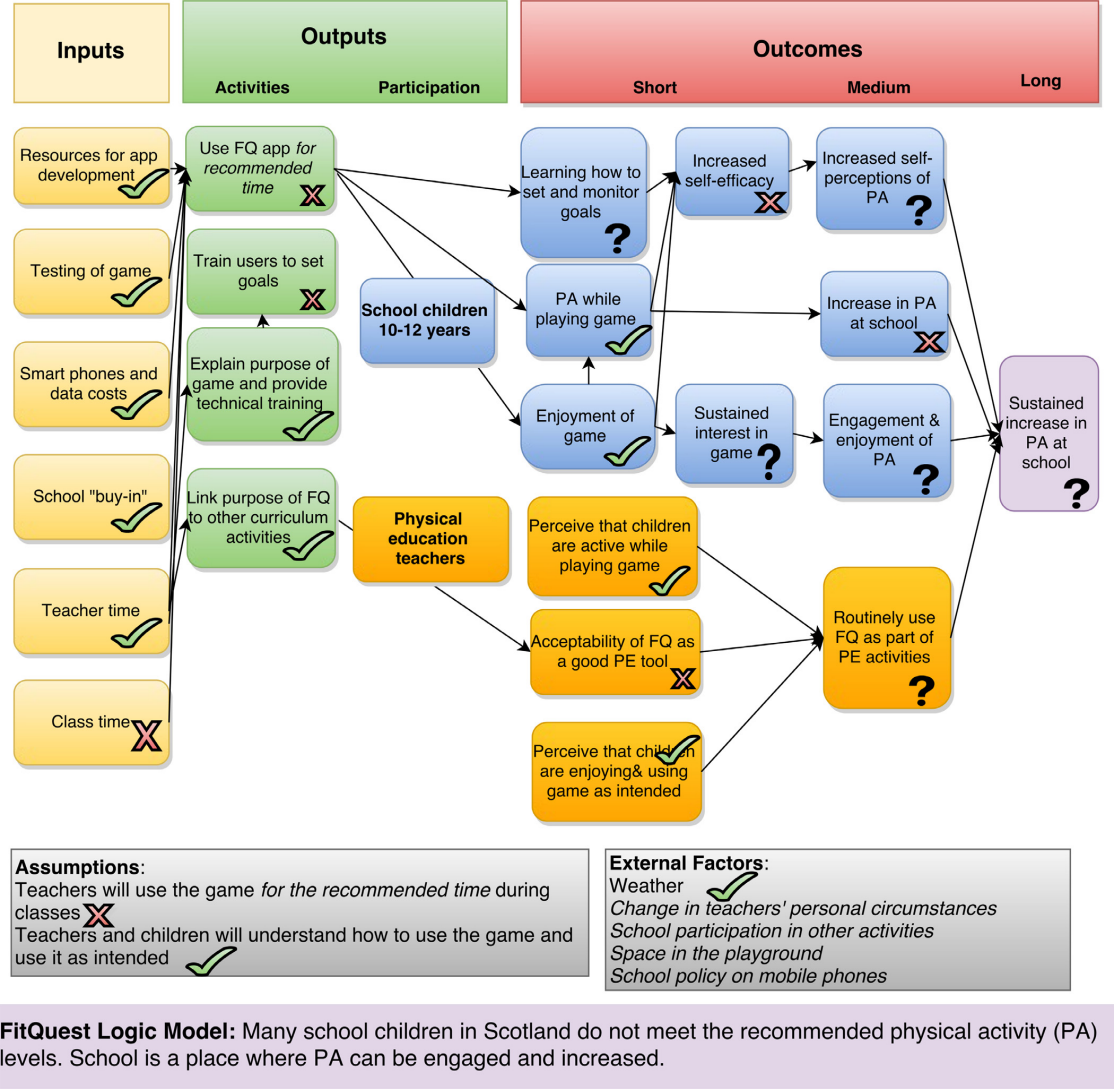
Revised logic model.

The initial logic model for the FitQuest project was refined as a result of the evaluation (see Fig 3).

Aspects of the model which worked as intended are annotated with a tick mark. Those which did not work as intended are highlighted with a cross, and those where the evidence is mixed, or as yet unresolved are indicated by a question mark. Changes to the text from the initial model are in italics.

#### Assumptions

Firstly, it is clear that assumption a) *that teachers will use the game during PE classes* was flawed. While all the intervention schools did use FitQuest on at least two sessions, on average the schools used FitQuest for only 35% of the recommended time (103 minutes over 5 weeks). The reasons for this included poor weather but also external factors such as teacher absence and competing school activities such as school trips, sports competitions, or tests. This is likely to have had a large impact on the success of the project–the success of PA interventions relies on time spent being active. Therefore, it is not possible to draw conclusions from the pilot RCT outcome measures about the efficacy of the FitQuest intervention as originally designed. The assumption b) that *teachers and children will understand how to play the game and play it as intended* held, and is clearly demonstrated in the interview, observation and log file data.

#### External and contextual barriers

As anticipated, the external factor of weather did have a negative impact on the intervention, both during the FitQuest sessions and in the post-test data collection. There was heavy rain during the post-test week for School 1 and School 7 which prevented the children from going out during the breaks. This is likely to have decreased their step counts because they had reduced opportunity to be active. In addition, changes in a teacher’s personal circumstances had a negative impact on the intervention. Disruptions to data gathering schedules and reductions in the number of sessions playing FitQuest were caused by life events of the teachers: illness, bereavement, and becoming a new parent. The participation of the school in other activities such as trips, competitions, and festivals disrupted and cancelled FitQuest sessions more often than anticipated. These findings are consistent with experiences from the deployment of the AHPC exergame in schools, which highlighted the important role of the teacher’s enthusiasm and ingenuity in supporting integration of the game into the daily routine[36].

Some schools (particularly 4 and 10) experienced technical difficulties in using the game due to poor or fluctuating GPS coverage in the playground. This caused some children to become frustrated with the slow update of their avatar’s position or unreachable game objects. Frustrations with technology in schools are common; the AHPC project also reported that both students and teachers found it hard to maintain enthusiasm when experiencing regular technology problems although in that case the glitches were related to malfunctioning pedometers rather than network coverage[36]. The issues of “seamfulness” (when the user becomes aware of “cracks” and “seams” in a service resulting from patchy network coverage, fluctuating signal strength, deviations in positioning) is a known issue in designing location based services[37]. To address this challenge, future designs of FitQuest could adopt the approach used in the design of the game Feeding Yoshi [38] or Can You See Me Now?[39] in which the game incorporates seamfulness positively as in integral design feature e.g. using patches of poor GPS cover as “hiding places” for players in a multiplayer game.

The phones were used at playtime in only one of the schools. Lack of space in the playground for some schools (e.g. School 10) made some of the teachers unwilling to allow the children to use the game during break time for safety reasons. This is in common with previous research on more traditional physical activity in schools e.g. [40] found that bigger playgrounds were associated with higher levels of PA.

A related barrier which we did not anticipate relates to school policies with respect to mobile phones in the playground. Some schools considered that allowing the use of borrowed phones at playtime for FitQuest would send an inconsistent message to the children who in general are banned from using mobiles phones in the school. While 35% of children of this age group in the UK have their own mobile phone[41], schools often do not allow them to be used on the premises [42] for a variety of reasons including perceived disruption to learning, equality of access, and fears regarding inappropriate use such as cyber-bulling. Some local education authorities in the UK are now experimenting with Bring Your Own Device (BYOD) policies which capitalise on the use of personal devices to support learning within an agreed framework of appropriate use. Indeed, the council with responsibility for the schools in this study is now considering implementing such a policy.

As identified by one of the teachers, the cost of a suite of mobile phones was considered prohibitive outside the scope of a research project at the time the study was carried out. However, this is already changing as school policies evolve to embrace the benefits of mobile devices for learning, with two main approaches: BYOD and school investment in large sets of tablet computers[43]. At a school with a BYOD policy in place, children could use FitQuest with their own mobile device (assuming it was ported for use with iPhones too) or a device borrowed from the school. For schools which have invested in suites of tablets, the design of FitQuest could be adapted to be used with larger tablet devices, as running with a tablet would be more cumbersome. For example, iBeacon technology could be used to transmit information from a badge on the child’s t-shirt to a tablet device positioned on a table. This would have the additional advantages that it would not depend on GPS, and would enable the game to be played indoors in poor weather.

#### Components of the intervention

Most inputs occurred as intended (apart from class time). The teachers received the intended training and discussion of how FitQuest links to the PE curriculum. As discussed above, although FitQuest was used during PE lessons, it was not used for the recommended time or number of sessions. Further, although we intended that the teachers should be active in training the children to set and monitor appropriate game goals, and encourage various forms of social play, this did not occur in general. This was often because the teachers were teaching the half of the class who were not currently using FitQuest. Those teachers who did find time to attend the FitQuest sessions occasionally seemed reluctant to interact with the children because they worried that it would *“*contaminate*”* the study. To capitalise on the emergent facilitator relating to the role of an interested adult, future projects might benefit from a FitQuest ‘champion’ for each class (similar to the Physical Activity champions in the Let’s Move! Active Schools initiative [44] a teacher, classroom assistant, or even an older pupil who encourages and discusses progress with the pupils. A study of peer PA promotion in a school context resulted in positive experiences for the children in role of leader as well as the younger children they encouraged[45], although the findings emphasise the importance of supporting the leaders with appropriate training. Involving older pupils as champions would suit the schools in this study, as they use a “buddying” scheme to promote positive social relationships among the older and younger pupils.

#### Outcomes

The qualitative findings suggest that most of the children enjoyed using the game. Existing questionnaire data from the series of previous studies during the design process indicated that children enjoy playing the game; the qualitative evidence from the current study indicate that the participants in this study also enjoyed it, particularly in the initial sessions.

There is some qualitative observational and self-report evidence that many of the children undertook moderate to vigorous PA during sessions. However, this did not translate to sustained interest in the game for all the children as planned (the evidence is mixed). There is qualitative evidence from interviews and observations that some of the children were able to appropriately set and monitor game goals, and that for some children achieving success in game goal setting was associated in gains in self-efficacy. Including guidance and appropriate default settings for goals would likely improve the game, and make this pathway more likely to occur.

In the view of the teachers, the children's PA was more vigorous during FitQuest sessions than other playground activities and compared favourably to other activity in other PE lessons. They also perceived that the children enjoyed the game and used it as intended. Some PE teachers had reservations about the acceptability of FitQuest as a PE tool, which may also have impacted on the amount of time they allocated to FitQuest in their classes and have a bearing on whether they would routinely use it as a regular part of PE activities in the medium term. Given this, it is worth considering whether the school context is most suitable for FitQuest, or how it could be adapted to meet the needs of teachers. Further work is underway to trial adapted versions FitQuest in family settings, and in the context of a healthy weight club for children organised by the National Health Service.

At this stage, the evidence for the proposed medium and long term outcomes in the logic model is negative or inconclusive, which may be a result of “broken links” in the assumptions, activities and short term outcomes. The failure of the wider intervention to impact PA is likely due to flaws in both the how the design of the software implemented the underlying programme theory and contextual factors. The qualitative evidence, considered with the findings reported in [11], suggest that the logic model pathways worked for some children (such as those with initially low self-efficacy, who enjoyed the game and set achievable goals) but not all. Examination of the aspects of the model which did not work as intended suggests ways in which the intervention could be redesigned to improve matters (e.g. by helping realistic game goal setting through default targets, introducing new content to sustain interest, capitalising on social play and possibly removing competition). It would also be beneficial to work more closely with teachers to help the technology to integrate better with the context (e.g. changing the hardware to use devices which are acceptable and established in school, and aligning it more closely with curricular goals in PE lessons).

#### Strengths and limitations of the study

This study has strengths which can shed new insights into the importance of context on the effectiveness of interventions. Firstly the intervention has a clearly articulated, and theoretically informed, programme theory. This enabled a nuanced analysis of the data to see where the programme theory needed revising. Secondly, the use of qualitative data (triangulated with log-file and questionnaire data) has enabled us to interpret the quantitative results and allowed modification of the programme theory. Without the qualitative insights, it would not have been possible to identify the crucial role that the school context made. Indeed, this exergame may have been consigned to the ever growing list of ineffective public health interventions. However, the qualitative insights suggest that further modification of the intervention may lead to an increase usage and more promising outcomes.

A potential limitation of the data collection method is that the researchers who designed the game also observed the sessions and interviewed the children and teachers. This could lead to bias or inhibit the participants from voicing their true opinions. However, this was mitigated by the team’s request to the participants for critique and suggestions about the game. As is clear from the transcripts in the supplementary materials, the children did in fact offer negative opinions about the game and were diligent in reporting problems with it to the researchers.

A critical case sampling approach was used for selecting the children to participate in the post-evaluation interviews. Using observational, log-file and survey data, researchers picked those cases most representative of the key research themes. One limitation of this approach is that it is prone to researcher biases, especially when compared to a random approach to sampling. However, clear criteria for selection, and utilization of a mixture of qualitative (observation) and quantitative (log-files and surveys) helped mitigate the risk of bias during subjective selection.

#### Recommendations

The results of this study are instructive at two levels: 1) informing how school-based exergame interventions could be improved in the future and 2) advancing methodologies for approaching the evaluation of complicated socio-technical interventions.

*Improvements in school-based exergame interventions*. From a technological point of view, exergames in schools are more likely to be successful if they incorporate opportunities for social interaction, particularly collaboration. However, the technology by itself will not have the desired impact if the teachers and wider school environment are not receptive. The perceived suitability of the game for PE classes and indeed the place of smart phones within schools are particularly important. It is likely to be more effective it the exergame is developed for a technology platform which is already commonly used in the school setting. Dialogue with the teachers on the most effective ways to work with the game from both pedagogical and logistical perspectives is also important.

*Evaluation methodologies of complex socio-technical interventions*. Systematic reviews of exergames have predominantly considered evidence from studies of commercial console games, with a focus on lab-based settings [8]. As developments in technology offer more flexibility to assist users in their everyday lives, our evaluation methodologies must likewise adapt. There are methodological challenges associated with studying technology interventions in real-world settings which must be addressed. There is a rich body of work in the human-computer interaction community which has developed appropriate methods for *designing* technology to understand the needs of real-world users. The Human–computer interaction (HCI) literature contains a decade of work on (often theoretically informed) technology designs for behaviour change across multiple domains[46]. However, work on robust methods for *evaluating* the effectiveness of behaviour change technology used at scale in complicated real life settings is at an early stage[46]. In order to exploit the potential benefits of new behaviour change technologies, technologists need to understand not only *whether* their designs worked for particular groups of intended users but *why*[47]. They also need to know *which* components of a potentially multi-faceted design are effective, so that they can build them into future designs. Beyond this, however, technology evaluations must carefully document the socio-technical context in which the technology is used because the real world can place many barriers to the successful use of even the best designed software. Similar observations have been made by those critiquing methodologies for complex RCTs within public health[16,17]. Future evaluation of exergames and behavioural change technology more generally would benefit from ongoing collaborations between HCI and public health researchers.

## Conclusions

The qualitative results reported here contextualise the findings of a pilot cluster RCT in 10 primary schools which failed to detect an impact of the FitQuest exergame on 10–11 year olds’ self-efficacy, post-test step counts or MVPA. While the underlying programme theory which focuses on making PA fun and the use of game goal setting as an approach to behaviour change appears to be appropriate, critical reflection on the findings suggests that the intervention could be more successful in future. by assisting children with realistic goal-setting. The game could also be improved by capitalising on social play through multi-player games, introducing a peer FitQuest “champion” and sustaining players’ interest by introducing new games or art assets periodically. Redesigning the game mechanics to integrate GPS glitches as part of the game may also be beneficial. There were contextual factors which meant that there were insufficient opportunities for the children to play the game for the recommended time. In addition to technical changes, the contextual barriers to the success of this exergame can be overcome by working with teachers to improve logistical aspects of working with the technology and consulting teachers on how the game could be integrated as part of PE pedagogy.

## Supporting Information

S1 Checklist Consolidated criteria for reporting qualitative studies (COREQ) checklist for FitQuest qualitative data. (DOCX)

S1 Transcripts (DOCX)
