# Supplementary material for: Understanding the Importance of Context: A Qualitative Study of a Location-Based Exergame to Enhance School Childrens Physical Activity
Source: PLoS One. 2016 Aug 22;11(8):e0160927. doi: 10.1371/journal.pone.0160927 (PMC4993470; doi:10.1371/journal.pone.0160927)
Supplement: S1 Checklist — (DOCX) [file pone.0160927.s001.docx]

S1 Checklist

Consolidated criteria for reporting qualitative studies (COREQ): 32-item checklist

This is the COREQ checklist which accompanies the qualitative data gathering analysis for this dataset. Authors are as follows:

Judy Robertson^1^

Ruth G Jepson^2^

Andrew Macvean^3^

Stuart Gray^1^

1. Moray House School of Education, University of Edinburgh, Edinburgh, Scotland, UK
2. Scottish Collaboration for Public Health Research and Policy, University of Edinburgh, Edinburgh, Scotland, UK
3. Computer Science, Heriot-Watt University, Scotland

| **No** | **Item** | **Guide questions/description** |
| --- | --- | --- |
| **Domain 1: Research team and reflexivity** |  |  |
| Personal Characteristics |  |  |
| 1. | Interviewer/facilitator | Which author/s conducted the interview or focus group?  First author, third author. The fourth author helped with observation sessions in addition to the 1^st^ and 3^rd^ authors and was present as an observer in the interviews at School 7. The 2^nd^ author was not involved in data collection. |
| 2. | Credentials | What were the researcher's credentials? *E.g. PhD, MD*  First, second and third authors have a PhD. The fourth author is a PhD candidate |
| 3. | Occupation | What was their occupation at the time of the study?  First author: Senior lecturer, second author: senior scientific advisor, third author: research fellow, fourth author: PhD student |
| 4. | Gender | Was the researcher male or female?  The first author and second authors are female, the third and fourth authors are male |
| 5. | Experience and training | What experience or training did the researcher have?  The authors have 15, 20, 4 and 1 year of experience as researchers. The 1^st^, 3^rd^ and 4^th^ authors are computer scientists, specialising in human-computer interaction for children. The 2^nd^ author is a public health researcher. The 1^st^, 2^nd^ and 3^rd^ authors have training and experience in qualitative methods. |
| Relationship with participants |  |  |
| 6. | Relationship established | Was a relationship established prior to study commencement?  The children met the researchers for the first time at the start of the study. At the time of the interviews, the participants were familiar with the researchers because they were present during observation sessions. |
| 7. | Participant knowledge of the interviewer | What did the participants know about the researcher? e*.g. personal goals, reasons for doing the research*  The participants knew that the researchers were from a local university, that the 3^rd^ and 4^th^ authors were supervised by the 1^st^ author and that the game was developed by the 3^rd^ and 4^th^ authors. The informed consent forms which they signed explained the purposes of the study. |
| 8. | Interviewer characteristics | What characteristics were reported about the interviewer/facilitator? e.g.  The children knew that as designers of the game, we intended that it should increase PA and that we hoped it would be fun. We told the children that we wanted their help in improving the software, and that we welcomed feedback. (They often gave us suggestions during the study and were not shy to be critical). |
| **Domain 2: study design** |  |  |
| Theoretical framework |  |  |
| 9. | Methodological orientation and Theory | What methodological orientation was stated to underpin the study?  We used thematic analysis to explore the programme theory |
| Participant selection |  |  |
| 10. | Sampling | How were participants selected?  We observed sessions at all five of the FitQuest schools at least once a week. All participants taking part on those occasions were observed. Author 1 observed at Schools 1,7,10. Author 3 observed at all intervention schools, and Author 4 at Schools 2, 4, and 7. Where possible, two observers were present at each session, and observers were matched to schools according to the convenience of timetabling and geographical location.  We interviewed a selection of children (12 in total) from two of the five schools who used FitQuest, one from each wave of the study (school 2 and school 7). These two schools were selected according to the availability of the classes during the post-test week. The children were selected by the researchers: the two researchers who observed the sessions at the school discussed which children displayed behaviour related to the aims of the study such as changes in self-efficacy. We wanted to use the interview questions to gain more insight into these behaviours than was possible from “snapshots” of interactions during the sessions. We chose an equal mix of boys and girls, and cases where we anticipated the children would have negative as well as positive views.  We interviewed 3 teachers from the intervention schools (2 from school 2 and one from school 10).All teachers were invited to be interviewed, but only these teachers were available. |
| 11. | Method of approach | How were participants approached? e*.g. face-to-face, telephone, mail, email*  The teachers were contacted by email to arrange the interviews. The child interviewees were approached by their teacher. |
| 12. | Sample size | How many participants were in the study?  In the wider RCT study in which this qualitative study took place, 115 were allocated to the control group, and 119 to the intervention. These children were observed in the sessions at which they were present. From these, a total of 12 children were interviewed. |
| 13. | Non-participation | How many people refused to participate or dropped out? Reasons?  In the wider RCT, 11 children allocated to the control group and 8 allocated to the intervention did not take part due to lack of parental consent. One child dropped out from the control group because her parent was not comfortable with the questions in The Children and Youth Physical Self-Perception Profile (CY-PSPP). At the advice of the institutional ethics committee, the use of this questionnaire was discontinued and the data collected to that point was discarded.  None of the children dropped out of the interviews. |
| Setting |  |  |
| 14. | Setting of data collection | Where was the data collected? e*.g. home, clinic, workplace*  Session observations took place in the school playground. In school 2, child interviews took place in an open plan area outside the classroom. In school 7, child interviews took place in the corner of a classroom. Teacher interviews also took place in classrooms during break time. |
| 15. | Presence of non-participants | Was anyone else present besides the participants and researchers?  For the observation sessions a member of school staff (a PE teacher, class teacher or classroom assistant) was present for supervision in addition to the researchers.  Only the researchers were involved in the interviews, although school staff were within sight and earshot. |
| 16. | Description of sample | What are the important characteristics of the sample? *e.g. demographic data, date*  The interviews took place in December 2013 and April 2014 during the post-test week. The children were 11 years old. The location of School 2 is in the second most deprived quintile of the Scottish Index of Multiple Deprivation. School 7 is in the second least deprived quintile. |
| Data collection |  |  |
| 17. | Interview guide | Were questions, prompts, guides provided by the authors? Was it pilot tested?  The authors prepared a set of questions for the semi-structured interviews in advance. It was not pilot tested. |
| 18. | Repeat interviews | Were repeat interviews carried out? If yes, how many?  There were no repeat interviews |
| 19. | Audio/visual recording | Did the research use audio or visual recording to collect the data?  The interviews were audio recorded. Observers either wrote notes by hand, or dictated their notes into an audio recorder during the session. The audio recordings contained some min-interviews with children. |
| 20. | Field notes | Were field notes made during and/or after the interview or focus group?  The researchers typed field notes after each observation session which contained a transcript of their hand-written or audio notes and reflections on the session. |
| 21. | Duration | What was the duration of the interviews or focus group?  20 minutes |
| 22. | Data saturation | Was data saturation discussed?  No |
| 23. | Transcripts returned | Were transcripts returned to participants for comment and/or correction?  No |
| **Domain 3: analysis and findings**z |  |  |
| Data analysis |  |  |
| 24. | Number of data coders | How many data coders coded the data?  Author 1 coded the child interviews and Author 3 coded the teacher interviews. Author 2 checked the coding. |
| 25. | Description of the coding tree | Did authors provide a description of the coding tree?  Yes |
| 26. | Derivation of themes | Were themes identified in advance or derived from the data?  They were derived from the logic model in advance. Emergent findings are also reported in a separate section |
| 27. | Software | What software, if applicable, was used to manage the data?  Dedoose. |
| 28. | Participant checking | Did participants provide feedback on the findings?  No |
| Reporting |  |  |
| 29. | Quotations presented | Were participant quotations presented to illustrate the themes / findings? Was each quotation identified? e*.g. participant number*  Yes, quotes are labelled by pseudonym, school ID and source (e.g. observation or interview) |
| 30. | Data and findings consistent | Was there consistency between the data presented and the findings?  Yes. The qualitative data is also used to make sense of the quantitative findings. E.g log file data is used to give context for quotes |
| 31. | Clarity of major themes | Were major themes clearly presented in the findings?  The major themes are clearly identified by section headings |
| 32. | Clarity of minor themes | Is there a description of diverse cases or discussion of minor themes?  Diverse cases (negative examples) are considered, for example the children who did not like FQ (Dominic) and children’s criticisms of the game. Minor themes (such as authentic learning) are mentioned. |
