# Supplementary material for: Understanding the Importance of Context: A Qualitative Study of a Location-Based Exergame to Enhance School Childrens Physical Activity
Source: PLoS One. 2016 Aug 22;11(8):e0160927. doi: 10.1371/journal.pone.0160927 (PMC4993470; doi:10.1371/journal.pone.0160927)
Supplement: S1 Transcripts — (DOCX) [file pone.0160927.s002.docx]

# S2 Transcripts

# A qualitative study of an exergame for children’s physical activity: interview and observation excerpts

This file contains excerpts from the interview excerpts and observations notes which support the reporting of the qualitative analysis. It is organised under the same section headings as the paper.

### Game goal setting and monitoring

*Researcher’s notes: “Ellen asks the researcher for goal advice. The researcher says “if you just got 20 points and we’ve got the same amount of time again, what’s a good goal?”. Ellen replies ‘30’”* [School 1, observation notes, Session 1].

***

*Researcher: “If you had successfully managed to get your 50 points, what would you do then?”*

*Zoe: “I would probably set it a couple of points higher, because if I knew I could reach that score, then maybe like, make it a bit harder for me to see if I could reach that score.” [Zoe, interview, School 2]*

*Isabel: “If I was not getting my goal, then I had set myself a harder goal that I couldn’t reach, then I would set myself a lower goal. Say I did 50 points, if I didn’t get that then I would try 40 points. Then I would see if I could improve.” [Isabel, interview, School 2]*

***

*Researcher: “What’s one thing you liked about the game?”*

*Lesley: “That it had the goals for it. That you could see how many points you were getting. When you just did a session you know what you achieved. And then altogether what you achieved.” [Lesley, interview, School 7]*

***

*Researcher: “What is your goal today James?”*

*James: “I dunno, to win games.”*

*Researcher: “How many games are you aiming to win?”*

*James: “Well I've won 26 before.”*

*Researcher: “Wow, so are you going to beat that?”*

*James: “Nah, I think I'll go for 5.”*

*Researcher: “Why 5 if your record is 26?”*

*James: “Because I want to make sure I reach my goal so I do 5 games at a time.” [School 4 observation, Session 6]*

***

Researcher: *“how did you feel you reached your goal?”*

*Saanjh: “I got really excited.”*

*Aakil: “I never got to my goal of top the leader-board.”*

*Saanjh: “I reached quite a few of my goals, but then I decided not to cheese [be overly happy / bragful] about it.”* [Interview, School 2]

***

Douglas [on achieving a challenging target]: “*Because that would be an accomplishment, you know, completing it. I thought I would try and complete level 10 about 10 times, just to see. I made it. I did it 11 times, it was really fun*. “[Douglas, Interview, School 7].

***

*Simon: ”Can you put me back on the leader-board please?”*

*Researcher: “Do you mind if I ask why?”*

*Simon: “Because I've got the most points.” [School 4, observation, Session 6]*

***

Colin: “*if you're not at the top people slag* [tease] *you a bit*” [School 4, observation notes, Session 2].

***

Researcher: “So is the leader-board something that is very important to you?”

Kevin: “Yes! I always beat Scott!”

Researcher: “So given that, are you only looking at specific people to beat?”

Kevin: “Yeah just Scott” [School 7, observation notes, Session 3].

***

*Researcher: “What’s your goal for today?”*

*Brendan: “To top the leader-board.”*

*Researcher: “For certain games or overall?”*

*Brendan: “Overall. I’ve still got a bit of way to go. I’ve got 263 but I need to get to 790.”*

*Brendan: “I’m going to try and beat Sue. I need 5 points and then I’ve got her! Oh wait I’m already above Sue, let’s try to get Jane then!” [School 7, observation notes, Session 3].*

***

Researcher’s notes: *“The pupil comfortably at the top of the leader-board, Evan, was notably less active today. Instead, he spent much of the session admiring his own points tally and observing the competition going on further down the leader-board”* [School 2, observation notes, Session 4].

***

Ruby: “Ooh I’m 16th on the leader-board!”

Researcher: “So what do you think of the leader-board Ruby?”

Ruby: “You need a lot of points for it.”

Researcher: “Is it important to you where you are on the leader-board?”

Ruby: “No.” [School 7, observation notes, Session 3].

### Self-efficacy

Donald: “*It made me feel really happy that I had done better than last time.*” [Edward, interview, School 7].

***

Lesley: “*I felt quite proud because I don’t normally run or do anything fit.”* [Lesley, interview , School 7].

***

Linda: *“Maybe a couple of times I didn’t reach my goal but I just tried harder to beat it. Because I wasn’t really down with myself but I would keep trying.”* [Linda, interview, School 7].

***

Caroline: “*It’s good for getting you active but you’d have to do it more often I think because some people in the class like me are not that fast at running so we don’t really like it. I’m trying and I’m getting used to it now because it is fun*” [Caroline, School 7, observation notes, Session 4].

***

Isabel: “*Well I didn’t run that often, but when I had the phone in my hand, I wanted to run because I wanted to catch the wolf*.” [Isabel, Interview, School 2].

***

Isabel: “[If I did not achieve my goal I would feel] *Quite sad because like, then I would feel like I was not that sporty when I didn’t manage to reach my goal. I would feel quite sad*” [Isabel, Interview, School 2].

***

Ruth: “*I don’t like the leader-board. If you’re not doing well it can knock your confidence down a bit*” [Ruth, School 7, observation notes, Session 4].

***

Class teacher: *“he only likes to play games where he is the best and will win, or at least think he's the best. However, with the game, the leader-board does not lie and because he wasn't first he decided to stop putting in effort as an excuse”* [Teacher, School 4, Observation notes, Session 2].

***

*Researcher: “So what are you thinking about FitQuest today?”*

*Bert: “I was really up for it until I had to run around the school.”*

*Researcher: “Are you just not in the mood for it today.”*

*Bert: “Not right now, but I’ll do it!” [School 7, observation notes, Session 3]*

### Physical activity

Researcher’s notes: “*Her play was intense bouts of running and then stopping quite regularly. She was pretty out of breath on her rest periods. She spent her rests congregating with friends, e.g. sitting on playground equipment. During one rest she challenged another girl to a race, but her friend wanted to try another game instead. She asked me once what Mystery Game was. I explained and she said she was too tired to try it, so I suggested Collect the Coins instead. At one point she responded to the prompt [on the FitQuest software] to rate her exertion level. She said 9, and in my view this was about right based on her audible breath. At one point close to the end session, Marianne went right to the bottom of the playground to get a long run for a race. At end of session I asked her what she thinks of FitQuest. She said ‘good but tiring’.” [School 7, observation notes, Session 1].*

***

Zoe: *“Whenever we finished a game we like huddled up and decided which game to play next. Then we came back and had a wee rest.”*

Researcher: *“So you liked to run about sometimes then have a huddle and a chat and then go back out?”*

Zoe:” Yeh for like two minutes and just decide what game to play now.” [Zoe, Interview, School 2]

***

Brendan said *“Escape the Wolf* [a mini-game] *is really good for my stamina, especially on level 10”* [School 7, observation notes, Session 3].

***

Researcher: “How are you feeling about FitQuest this week compared to previous weeks?”

Douglas: “Well the first time I tried it, I wasn’t really good at it because I only got like 22 points but then I’ve started to improve so I think that it’s helped.”

Researcher: “Good. So now that you are better at the game, how does that make you feel?”

Douglas: “Well I feel like I can definitely run a lot longer than I used to be able to. I definitely think my stamina has improved and so has my speed!”

Researcher: “So is that important, that it has helped your fitness?”

Douglas: “Yeah that’s important because I play football, which is my number 1 thing.” [School 7, observation notes, Session 4]

***

Linda: “*I think I’m getting much faster with my running. I’m definitely pushing myself to do lots of running*.” [Linda, School 7, observation notes, Session 4].

***

Researcher’s notes: “*Instead of playing the game Martin and some of the other boys would do their own thing, only resuming play when they knew that they were being watched by the class teacher*”. [School 4, observation notes, , Session 3].

### Enjoyment of game

Child: “*It's really good, I'm going to play at break and lunch too*” [School 2, observation notes, session 1].

***

Douglas: *“it was a lot of fun. I ran about like crazy. It, yeh, it was awesome.” [Douglas, interview, School 7].*

***

Zoe: *“I think it’s good, because it gets me very out of breath”* [Zoe, interview, School 2]

***

Saanjh: *“It was also, it was really good, and it made, it is good exercise. As its purpose is exercise, isn’t it? It does that really well.”* [Saanjh, interview, School 2].

***

Dominic: “I don't like FitQuest, it's boring!” [Dominic, School 2, observation notes, session 2].

***

Researcher’s notes: *“Lachlan is complaining about coins generated out of area and he is confused about navigation. The researcher shows him dynamic camera. He seems grumpy about the game and has been complaining it is not proper PE.”* [School 10, observation notes, session 2].

### Sustained interest in game

PE teacher: *“I would say towards the end it* [motivation] *was a lot less. Initially it was huge, the first two weeks, it was great”* [PE Teacher, interview, School 2].

***

Maurice: “Well we liked it at the start, but we always knew where the sheep or where the chicken would go, so it got a bit boring.” [Maurice, interview, School]

***

Aakil: *“They were only actually enjoyably for the first few weeks, because then they actually got boring, a bit boring, after, you know we had played them a lot of times. There weren’t too many options really.”* [Aakil, interview, School 2]

***

Researcher’s notes: *“As we arrived for the class just after break time, many of the kids had just returned from playing the game. The result was a diminished desire to play games by many pupils. Despite this, there were still many highly motivated kids, especially those near the top of the leader-board who relished any opportunity to earn points”* [School 2, observation notes, Session 2].

***

PE teacher: *“I think once they had got used to it a little bit, maybe then it began to tail off”* [PE teacher, interview, School 10].

***

PE teacher: *“I was impressed by the children who kept at it that day. I thought one of the boys, Craig, he has been a bit lazy this year I would say, and he was really, he really kept on task the whole time”*. [PE teacher, interview, School 10].

***

Researcher: “How are you feeling about FitQuest now in comparison to previous weeks?”

Brendan: “Brilliant! It’s brilliant! It’s brilliant. I’m still loving it!”

Bert: “It’s really good! I’ve got a stitch from how hard I’ve been working.” [School 7, observation, Session 4].

***

Linda: *“I’m enjoying it more because I’m finding it easier as the weeks go on”* [School 7 observation, Session 4].

***

Robert: *“It’s better* [now] *because we know what we’re doing”*.

***

Linda*: “At the start I just ran anywhere because I didn’t know what to do. But then as the weeks went on I felt it was easier for me because I knew how to change the camera settings.”* [Linda, interview, School 7].

***

Emma: *“once you got* [understood] *the game it just got more and more fun and it got more easy”* [Emma interview, School 7].

### Teachers’ opinions about FitQuest as a tool for PE

PE teacher: *“When you saw them playing with the phones they were absolutely belting it as fast as they could …I think the phones were 30 seconds of very strenuous physical activity, running as fast as you could. Followed by a minute of idling and recuperating, getting your breath back and then on to the next bit, so I think those two things there, this idea of really strenuous PA is good”* [PE teacher ,interview, School 2].

***

PE Teacher *“I think it is very good for their fitness. I think they ran much more than they maybe would do if you told them to go and run round with a friend for 10 minutes, they wouldn’t do that.”* [PE teacher ,interview, School 10].

***

PE teacher: *“I* [was] *immediately sceptical about it. I think that* [technology] *is wonderful, but it has a place. I think it has to be really good or you shouldn’t be using them in PE. But this isn’t PE. This is about playground, and is tapping into what kids are doing normally. Anything that can get them energised and using things that they’ve already got in their pocket... So, I think the idea is good. Progressive. I think it is more to do with physical activity than physical education, but I am very happy for you to prove me wrong and for you to bring it in. I’m very happy for you to have come and done the project. It has been very good.”* [PE teacher, interview, School 2]

***

*PE teacher: “Well I felt, you know, they were looking at the mobile phone, and there wasn’t enough interaction amongst the children, unless at the very end when they swapped over they were saying ‘I was a certain place* [on the leader-board]*. So there was no teamwork to it, and there was no social aspect to it, and I think that’s quite nice to have in PE and important in Curriculum for Excellence* [the Scottish curriculum] *that we have just now. It is important that there is a lot of discussion and things, where as they are just getting the hand held phone and they are away, you know.”* [PE teacher, interview, School 10]

***

Class teacher: “*To be honest it kind of ran itself, because they are kind of a self-motivated class, and they took responsibility themselves. Someone counted the phones in at the end and things, they made sure they were charged up*”. [class teacher, interview, School 2]

***

PE teacher School 10: *“I couldn’t see that I would use it for a block of work, I think it would be much more a, ‘right, we’ve done that block of gymnastics, we have 2 weeks until the end of term, the weather is not as good as we would like for what we’re doing, let’s have a go at this* [FitQuest]*. Or maybe we would fit it into fitness blocks. So you know you might do a circuit one week, aerobics the next, and then we might do that* [FitQuest] *as well.”* [PE teacher, interview, School 10]

### Teachers’ perceptions of children’s enjoyment

Class teacher:: *“It’s a really good project to involve the children with. I think particularly at the start, they were really enthusiastic about it”* [Class teacher, interview, School 2].

***

PE teacher: *“That type of immediate feedback, children really like that. You know, whether it is timing them over a distance, or points on a leader-board, I think they like to see their efforts, do make success, they did score points, and so I think that was a good thing about the game too.”* [PE teacher , interview, School 10 ].

## Inductive (Emergent) findings

### Suggestions for improving the game

*PE teacher: “Maybe I could have lead a theme for each lesson each time they got the phone out. Maybe it was a new game, maybe it’s the leader-board, how to change the difficulty settings. Maybe we could have started off with that as a starting point, rather than just handing out the phones and saying ‘go play’.”* [PE teacher, interview, School 2]

***

*PE teacher: “I think the idle period would be less if everyone started of ‘here, and you do that and you go to this bit’ and then there is like 20 seconds and then it loads it for you and then it is like ‘go and you have to do this’. You get your breath back while the game updates itself, gives you a bit of feedback, sets you a new challenge. It’s* [currently] *all at the kids’ pace, whereas maybe you could make it all at the apps pace, take them through it a bit quicker.”* [PE teacher, interview, School 2]

***

*Saanjh: “Yeh, if they lose, decrease by one point. Or decrease by whatever points based on how they ran, or if they got caught really easily, like if they weren’t even moving at all and just being too lazy, then you could decrease 8 points and they would be like ‘woo, what, I better try again’.”* [Saanjh, interview, School 2]

***

Class teacher:  *“I found that children who tend to play in a more solitary way really enjoyed taking the phones out...I think just because, it gave them a sense of purpose in the playground. We do put out lots of things, playground equipment and things, but sometimes these kids need a bit more guidance. With FitQuest, it was all there for them. They had a bit of a challenge; they had in a way someone you were playing against because you had the NPCs [non-player characters], so I think in that sense that is why they enjoyed it.”* [Class teacher, interview, School 2]

***

Bert: “*design a more polished user interface*”

***

Eric : “*Make the GPS tracking thing faster to update. Sometimes you are in one place 10 metres from where the person is and it is annoying*.”

***

Aakil: *“Competitions and teams and things like that. So you link the internet together and make teams to go against others. Like, which team can, like for example with escape the wolf, you could do ‘which is the last man standing and on which team?’”.* [Aakil, interview, School 2]

***

*Brendan: “I’d like to see something where you could choose where you could come in the leader-board. Get into the top 10, get into the top 15. Get in front of someone in the leader-board.“*

***

*Researcher: “A particular person?”*

***

*Brendan: “Yes, if you want to get in front of a particular person you can try and do that”*

*[Brendan, interview, School 7]*

### Contextual factors

No specific excerpts relate to this category, as these emergent findings relate to logistical difficulties encountered by the research team rather than remarks by respondents.
